# Supplementary material for: Tandem-genotypes: robust detection of tandem repeat expansions from long DNA reads
Source: Genome Biol. 2019 Mar 19;20:58. doi: 10.1186/s13059-019-1667-6 (PMC6425644; doi:10.1186/s13059-019-1667-6)
Supplement: Supplementary file 1 — Supplemental methods, results, figures, and tables. (PDF 9545 kb) [file 13059_2019_1667_MOESM1_ESM.pdf]

Tandem-genotypes: Robust detection of tandem repeat expansions from long DNA reads

Satomi Mitsuhashi [1,*†], Martin C Frith [2-4,*†], Takeshi Mizuguchi [1], Satoko Miyatake[1], Tomoko Toyota [5], Hiroaki Adachi [5], Yoko Oma [6], Yoshihiro Kino [7], Hiroaki Mitsuhashi [8], Naomichi Matsumoto [1]

1. Department of Human Genetics, Yokohama City University Graduate School of Medicine

2. Artificial Intelligence Research Center, National Institute of Advanced Industrial Science and Technology (AIST)

3. Graduate School of Frontier Sciences, University of Tokyo

4. Computational Bio Big-Data Open Innovation Laboratory (CBBD-OIL), AIST

5. Department of Neurology, University of Occupational and Environmental Health School of Medicine

6. Department of Liberal Arts, Faculty of Medicine, Saitama Medical University

7. Department of Bioinformatics and Molecular Neuropathology, Meiji Pharmaceutical University

8. Department of Applied Biochemistry, School of Engineering, Tokai University

† These authors are contributed equally

* Corresponding authors:

Martin C Frith, PhD

Artificial Intelligence Research Center

National Institute of Advanced Industrial Science and Technology (AIST)

2-3-26 Aomi, Koto-ku, Tokyo, 135-0064, Japan

Telephone: +81-3-3599-8001

Fax: +81-3-5530-2061

E-mail: mcfrith@edu.k.u-tokyo.ac.jp

Satomi Mitsuhashi, MD, PhD

Department of Human Genetics

Yokohama City University Graduate School of Medicine

Fukuura 3-9, Kanazawa-ku, Yokohama, 236-0004, Japan

Telephone: +81-45-787-2606

Fax: +81-45-786-5219

E-mail: satomits@yokohama-cu.ac.jp

Supplemental Results

Low coverage plasmid tests

For human genome-wide sequencing, it is difficult and costly to obtain deep coverage such as 1000X, as we did for the plasmids in Figure 2. Also, for some repeat expansion diseases such as polyglutamine disease, the disease-causing copy number change threshold is usually less than 100.

Thus, we tested the ability of `tandem-genotypes` to distinguish small copy number differences in these plasmids with lower coverage. We randomly picked 50, 30 and 15 reads from each dataset, and compared the copy number predictions. Even with low coverage (15X) it is not difficult to distinguish copy numbers 18, 30, 70 and 130 for CAG repeats; 15 and 109 for CAA repeats; 21 and 52 for GGGGCC repeats (Additional File1: Figure S2).

Chimeric reads with exact numbers of repeats

As these plasmid-origin repeats were expected to vary because of nanopore base-call error or replication error in *Escherichia coli* during plasmid

preparation, we also made chimeric sequences by inserting the exact number of exact repeats, to test the accuracy of `tandem-genotypes`. The results were improved in all cases (Additional File1: Figure S3), in particular the *C9orf72* case with large strand-bias, indicating that deviations from the projected copy numbers are due to nanopore sequencing errors or replication error, and not systematic errors of `tandem-genotypes`.

PacBio No-Amp Targeted sequencing of *HTT* repeat expansion

If we wish to genotype a specific tandem repeat, a promising recent approach is "No-Amp Targeted sequencing" in combination with SMRT (single molecule, real-time) sequencing [1]. This is promising because it avoids error-prone DNA amplification. Thus, we tested whether our method is useful for this kind of data.

We used a recently-published set of DNA reads from eleven human individuals, targeting the huntingtin (*HTT*) locus [1]. The true repeat lengths are believed to be known +-1 copy, by fragment analysis [1].

Upon aligning these DNA reads to the reference genome (hg38), we observed that they are much more accurate than raw nanopore or PacBio reads: as expected, because these are CCS (circular consensus sequence) reads. The heuristics in `tandem-genotypes` may not all be relevant for these higher-accuracy reads. CCS reads can be used when a library insert is short, up to around 5 kb [1], so may not be applicable to large repeat expansions.

The HTT locus has a compound tandem repeat, with poly-CAG (encoding poly-Gln) next to poly-CCG (encoding poly-Pro). For this test, we ran `tandem-genotypes` with the reference genomic coordinates of the poly-Gln region (21 codons, whereas Höijer et al. regard the repeat as 19 codons. The boundaries are debatable.) For each sample, `tandem-genotypes` shows 2 sharp peaks (2 alleles), but some are a few copies off from the results of fragment analysis (Additional File1: Figure S20a). By referring to Table 1 of [1], it appears that these `tandem-genotypes` peaks agree perfectly with the length of the whole compound repeat, not just the poly-Gln part. This is not surprising, because `tandem-genotypes` counts expansions up to 60 bases

either side of the annotated repeat (see the main text). On the other hand, if we run it with option `--near=0`, it shows the length change of the poly-CAG region, in perfect agreement with fragment analysis (Additional File1: Figure S20b).

In general, there is no simple way to choose the repeat boundaries and/or the `--near` parameter. For less well-studied repeats, it may be unclear whether the length change of a whole compound repeat, or perfect sub-repeats, is more important. The default `--near` value is intended to avoid false negatives (which is important for clinical screening), by catching expansions that are aligned slightly beyond the repeat annotation. This is probably the main parameter that a user may consider adjusting.

Prioritization of copy number changes with random parameters

We tested random gene annotation scores (defaults +-50%) for multi-dataset prioritization (Additional File1: Table S5). We did not see drastic differences (Additional File1: Table S6), and all repeat expansions were ranked

very near the top. Among those parameters, the default parameters cope well with both long (~900 copies) intronic repeat expansion in the BAFME patient, and short (~100 copies) repeat expansion in protein-coding regions.

Supplemental Methods

Analysis of PacBio No-Amp dataset for *HTT* repeat expansion

These No-Amp datasets were downloaded from: https://github.com/NationalGenomicsInfrastructure/HTT-repeat-analysis. LAST alignment to GRCh38 and `tandem-genotypes` predictions for the Huntington's disease repeat locus were done like this:

```
last-train -P8 GRCh38 all.reads.fasta > train.out
lastal -P8 -p train.out GRCh38 each.reads.fasta | last-split > alns.maf
tandem-genotypes --near=0 hg38-disease-tr.txt alns.maf
```

We ran `last-train` just once, on all samples pooled, rather than running it separately on each sample. We did so because these samples are small and highly redundant.

Comparison to SV-calling software

`NGMLR-v.0.2.4` and `sniffles-v.1.0.8` were run like this:

`ngmlr` -t 8 -r GRCh38.fa -q file.fasta -x pacbio -o out.sam

`samtools` were used for sam-to-bam conversion, sorting and indexing, then:

`sniffles` -m out.sorted.bam -v out.vcf -s 3

For `PBSV-v.2.0.1` (https://github.com/PacificBiosciences/pbsv), we took the preceding `NGMLR` alignment file, then used these commands according to the `PBSV` instructions:

`pbsv` discover out.sorted.bam out.svsig.gz

`pbsv` call GRCh38.fa out.svsig.gz out.var.vcf

`NanoSV` (downloaded from https://github.com/mroosmalen/nanosv on May 2017) was run as follows on the LAST alignment output.

`maf-convert` -d sam out.maf > out.sam

Then, sam-to-bam conversion, sorting and indexing were done using samtools.

`nanosv.pl` -sambamba path-to-sambamba -c 3 out.sorted.bam > out.vcf

For these SV tools, we defined a tandem-repeat alteration to be "detected" if any kind of variant was predicted overlapping the tandem repeat annotation, else "not detected".

Prioritization of copy number changes with random parameters

We tested multi-dataset prioritization with 10 different random parameters (Additional File1: Table S5) like this:

`tandem-genotypes-join` --scores random-score file data1 : data2

References

1. Höijer I, Tsai YC, Clark TA, Kotturi P, Dahl N, Stattin EL, Bondeson ML, Feuk L, Gyllensten U, Ameur A: Detailed analysis of HTT repeat elements in human blood using targeted amplification-free long-read sequencing. Hum Mutat 2018, 39:1262-1272.

Figure S1

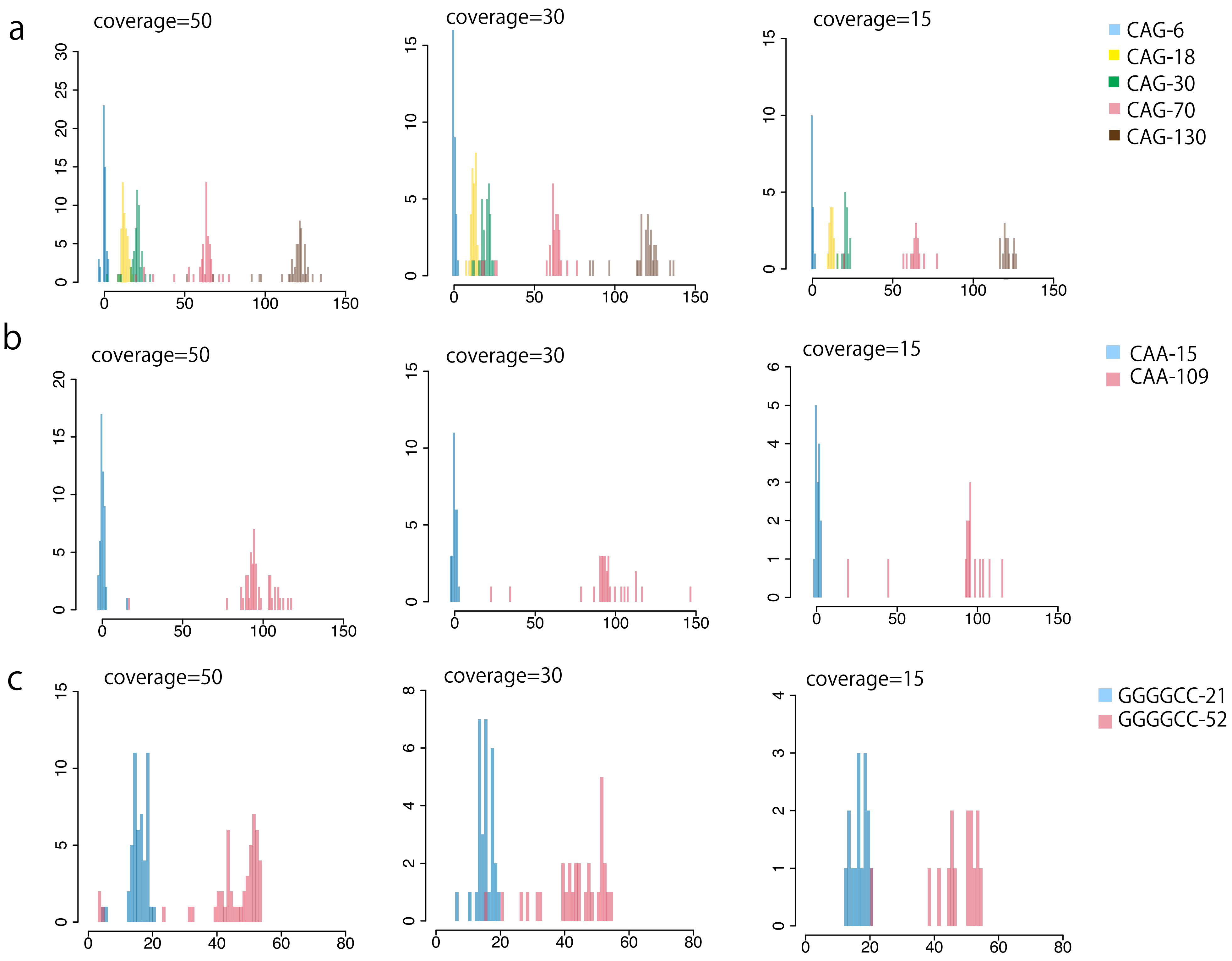

Figure S1

Distribution of predicted change in repeat copy number, for random samples of plasmid nanopore reads. Coverages 50, 30 and 15 were tested. (a) CAG repeats. (b) CAA repeats. (c) GGGGCC repeats. y-axis: read count, x-axis: change in copy number relative to the reference plasmid.

Figure S2

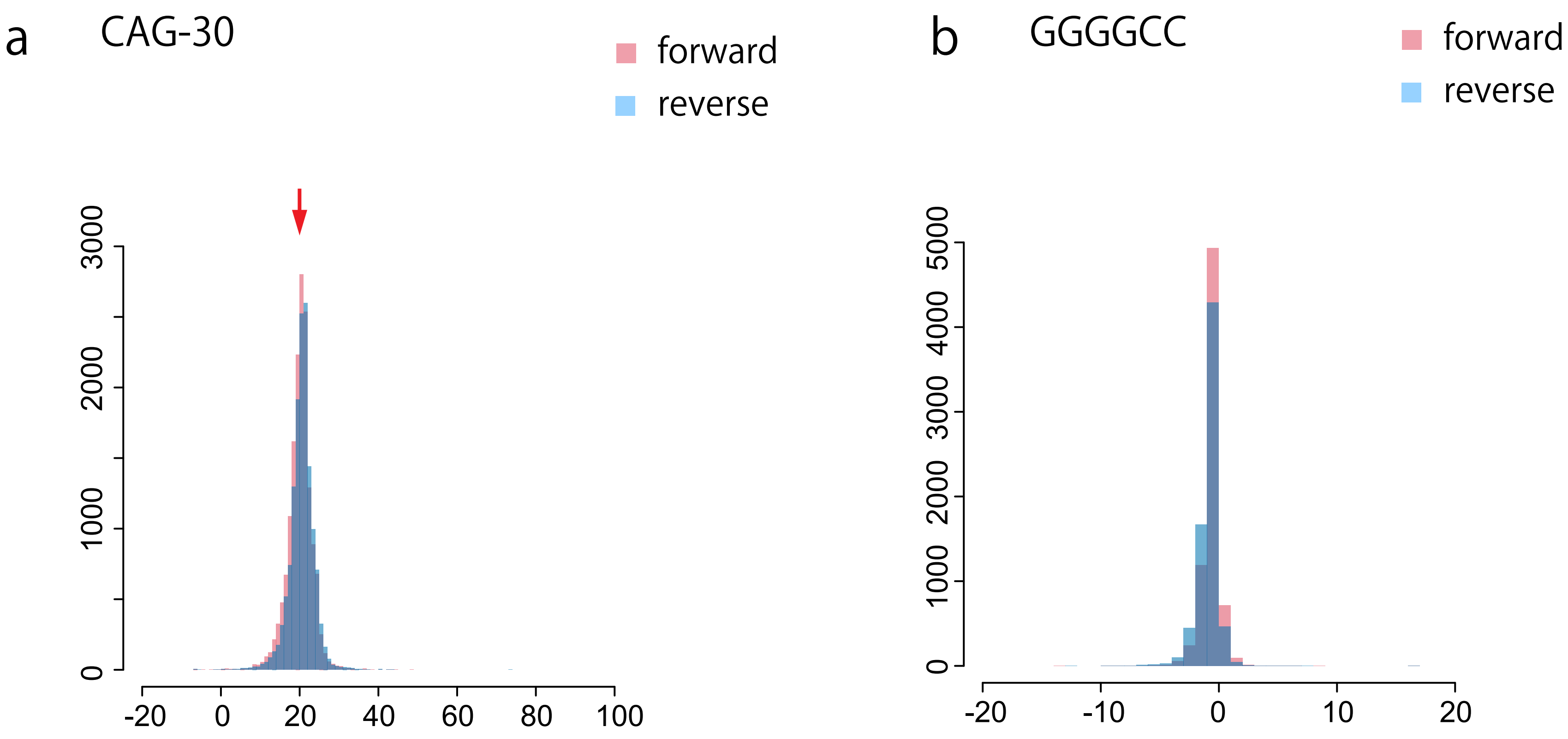

Figure S2

(a) Distribution of predicted change in repeat copy number, for nanopore reads from plasmid CAG-30 using a different restriction enzyme, DraIII. Unlike Figure 1c, the enzyme cut site in this dataset is distant from the start of the repeat (495bp and 2488bp). Forward (red) and reverse strand reads (blue) are depicted separately. y-axis: read count, x-axis: change in copy number relative to the reference plasmid. Red arrow: projected copy number change. (b) Genome-wide distribution of predicted copy number change of GGGGCC repeats relative to the reference human genome, for human MinION nanopore reads (rel3). Forward (red) and reverse strand reads (blue) are depicted separately.

Figure S3

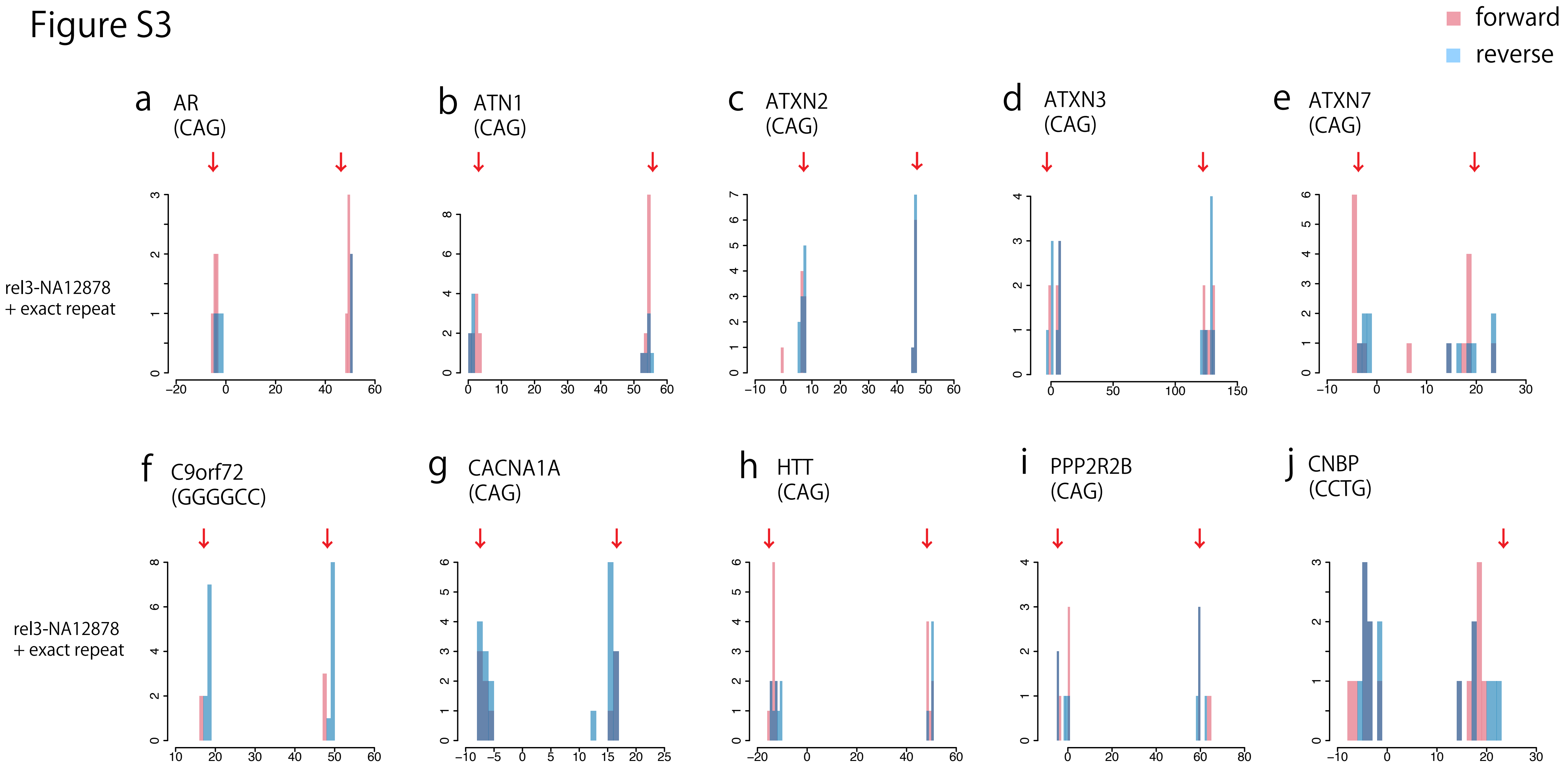

Figure S3

Distribution of predicted change in repeat copy number, for nanopore reads of human DNA with inserted exact repeats. Reads covering each of ten disease-associated repeat loci were selected, and the repeat region in each read was replaced by the exact repeats. y-axis: read count, x-axis: change in copy number relative to the reference human genome.

Forward (red) and reverse strand reads (blue) are shown separately. Red arrows: projected repeat copy changes.

Figure S4

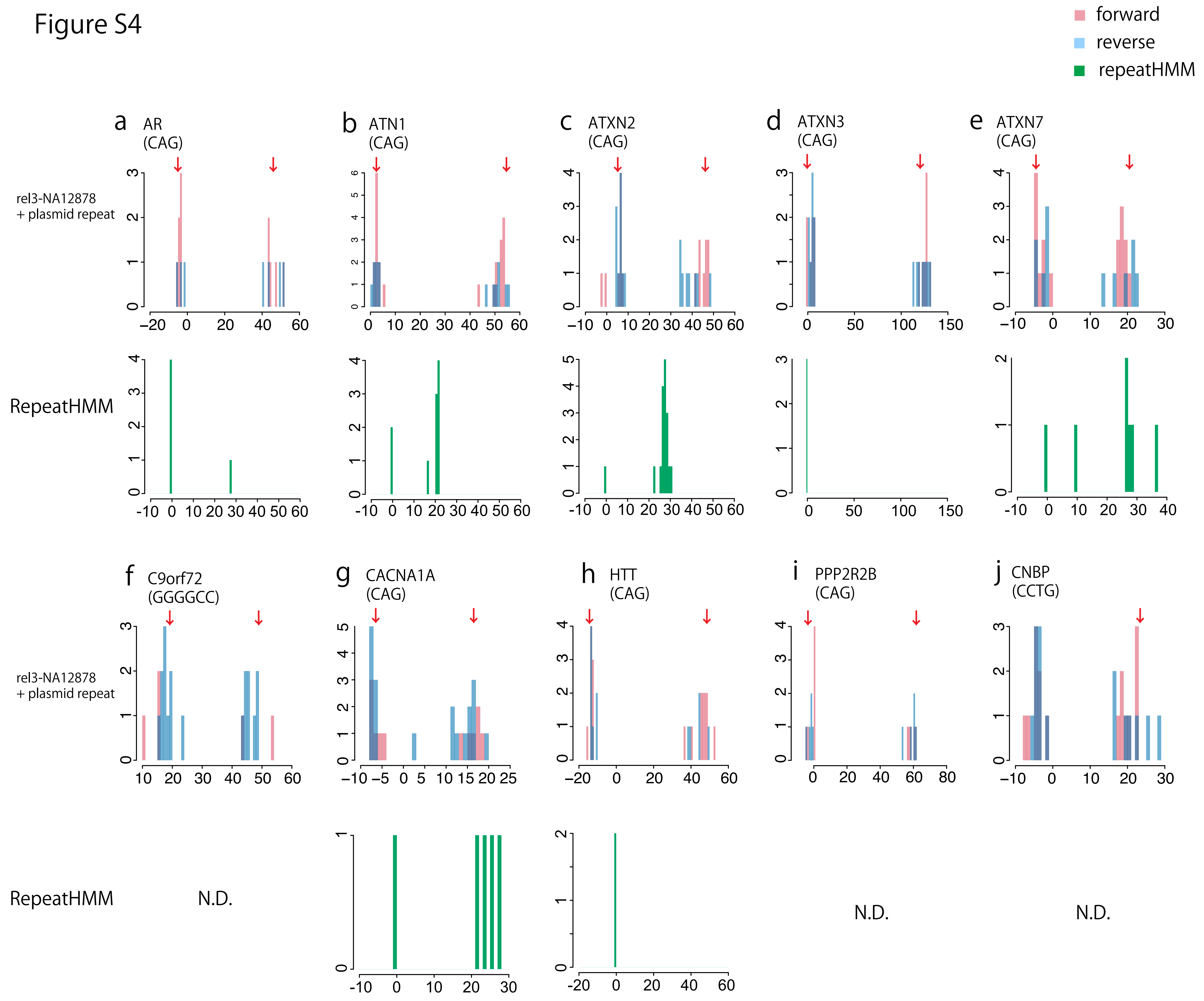

Figure S4

Distribution of predicted change in repeat copy number, for nanopore reads of human DNA with inserted repeats from plasmid nanopore reads.

Results from tandem-genotypes output (a-j top panels) were compared to RepeatHMM output (a-j bottom panels, shown green histogram). Red arrow: projected repeat copy number. y-axis: read count, x-axis: change in copy number relative to the reference human genome. Forward (red) and reverse strand reads (blue) are shown separately.

N.D.: not detected.

Figure S5

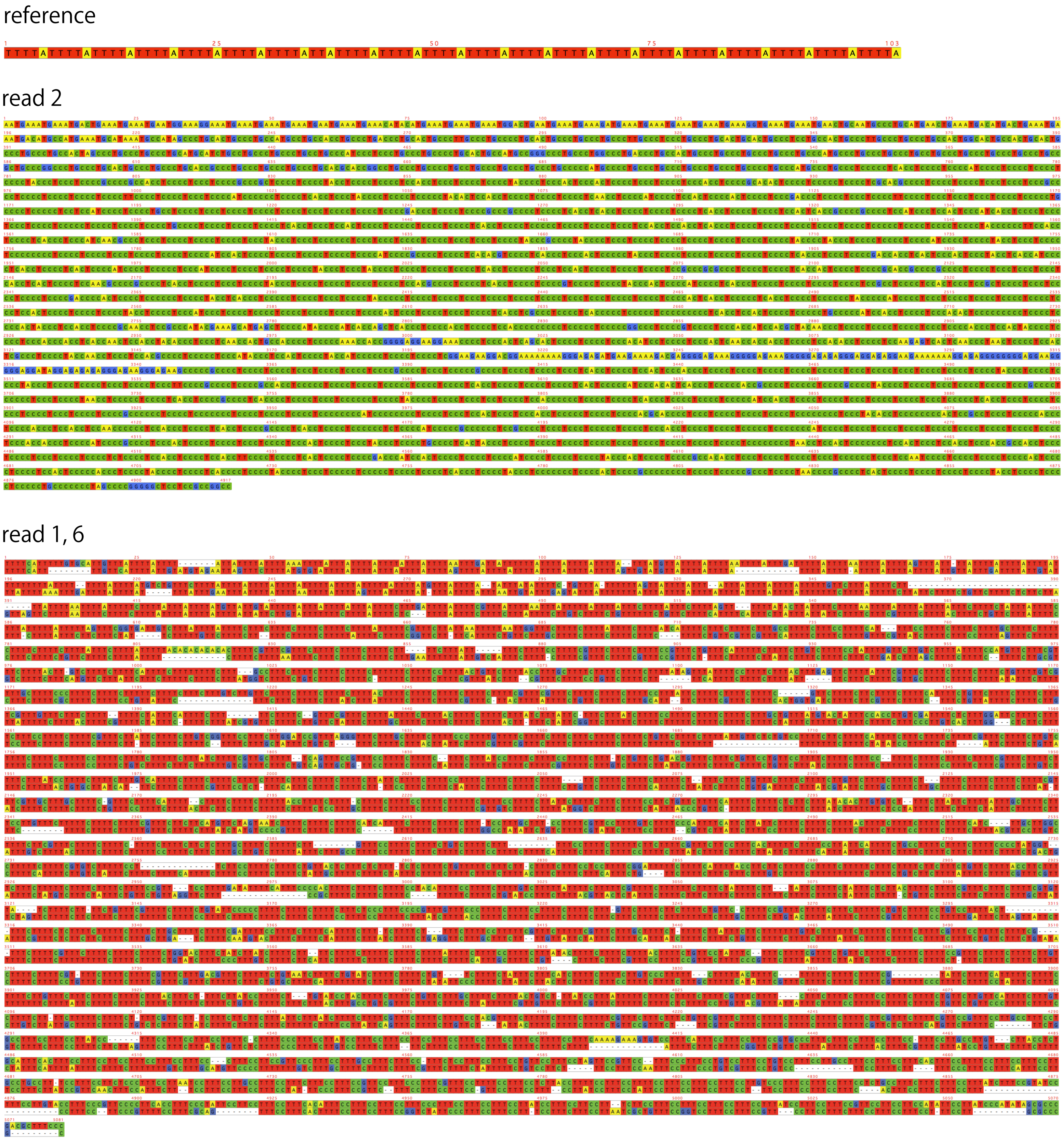

Figure S5

Raw reads from PacBio Sequel. Reads 1 and 6 were aligned by MUSCLE[26]. The expanded repeat appears to be heterogeneous, with several repeat units (TTTTA~100, TTTTC~600, TTTCC~100).

Figure S6

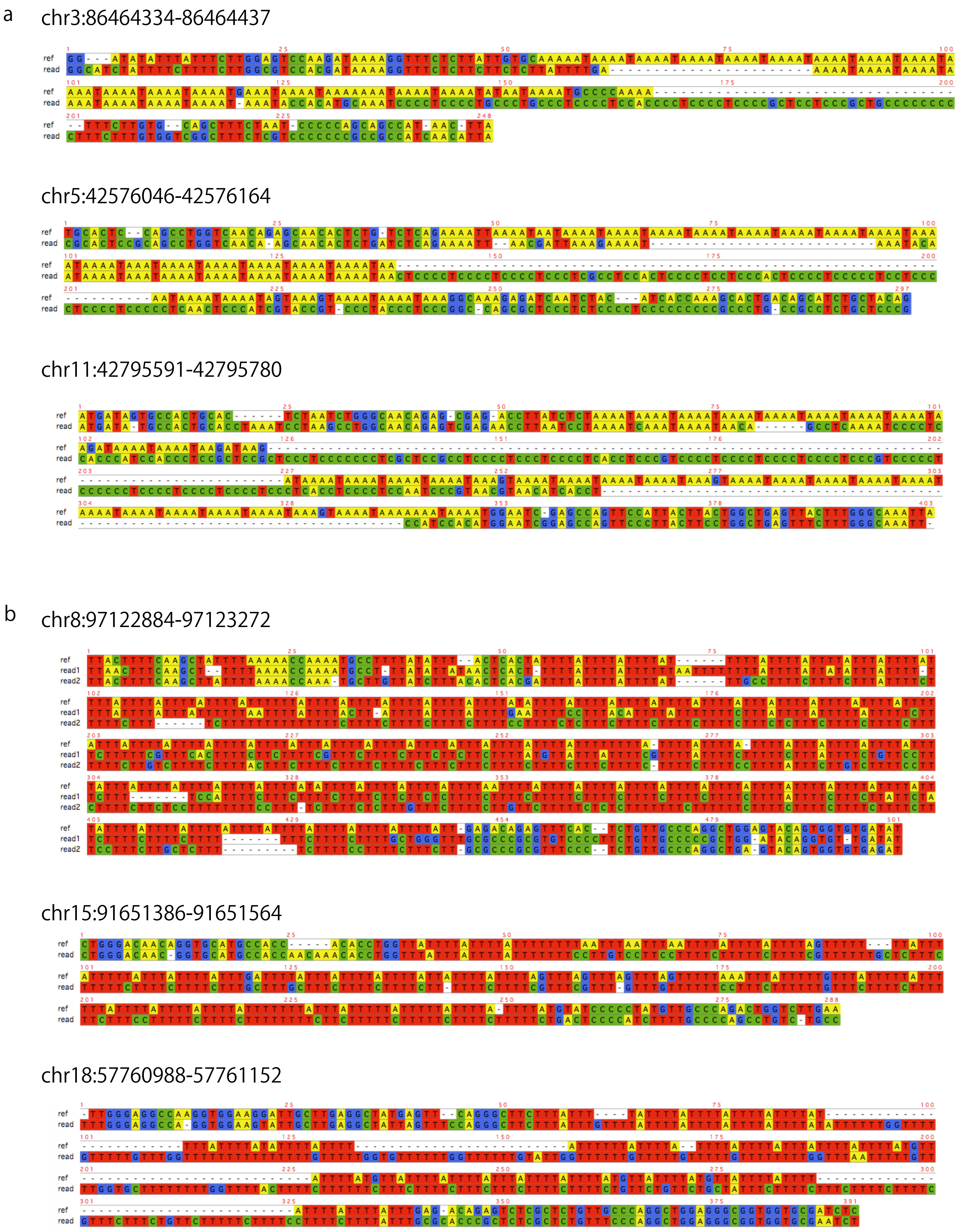

Figure S6
Examples of systematic error of PacBio reads in ‘TTTTA/AAAAT’ repeats. ‘AAAAT’ is read as ‘CCCCT’ (a) and ‘TTTTA’ is read as ‘TTTTC’ (b). Note that other reads at these loci did not show this kind of systematic error.

Figure S7

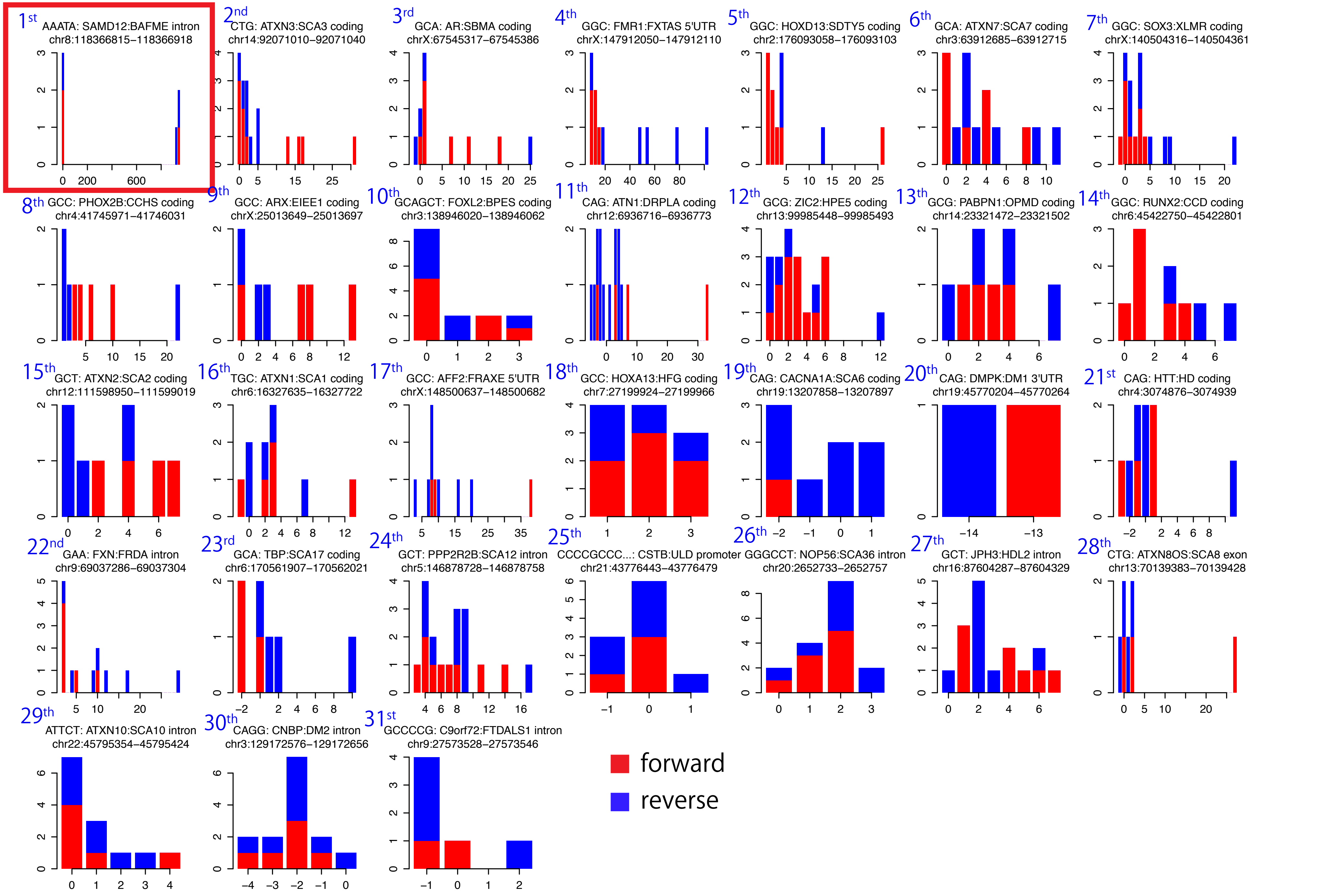

Figure S7

Changes in disease-associated repeats of a BAFME patient. The SAMD12 locus (red border) shows clear expansion of around 900 copies, and is prioritized 1st among 31 disease loci. The other loci have no obvious pathogenic expansions. Forward (red) and reverse strand reads (blue) are shown separately. These histograms were made by tandem-genotypes-plot.

Figure S8

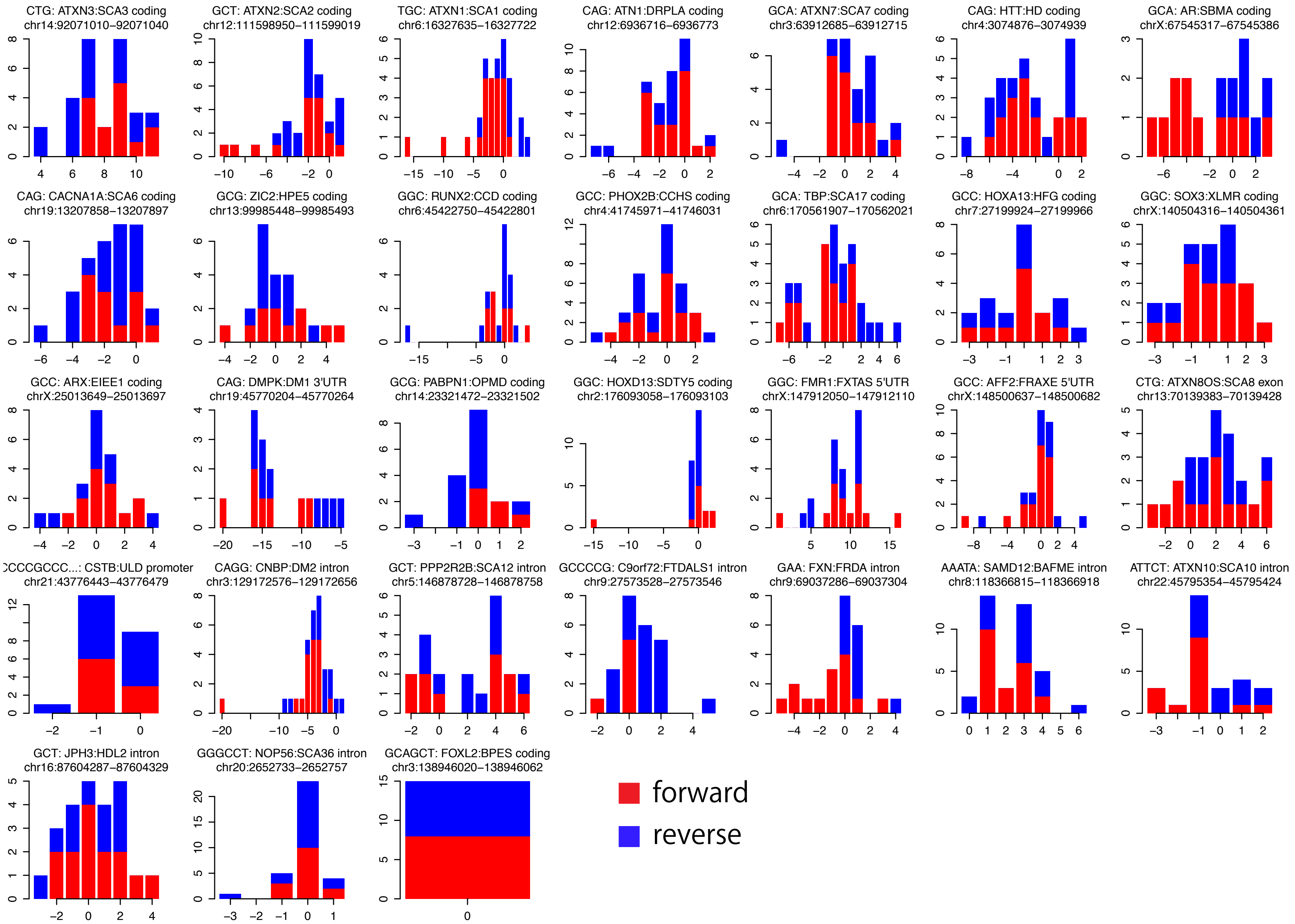

Figure S8

Disease-associated repeats in nanopore sequences for NA12878 (rel3). No obvious pathological expansions were predicted and peaks are around zero except *ATXN3*(~8), *FMR1*(~10), *DMPK*(~-15), and *CNBP* (~-5). Forward (red) and reverse strand reads (blue) are shown separately. Histograms are raw output of tandem-genotypes-plot.

Figure S9

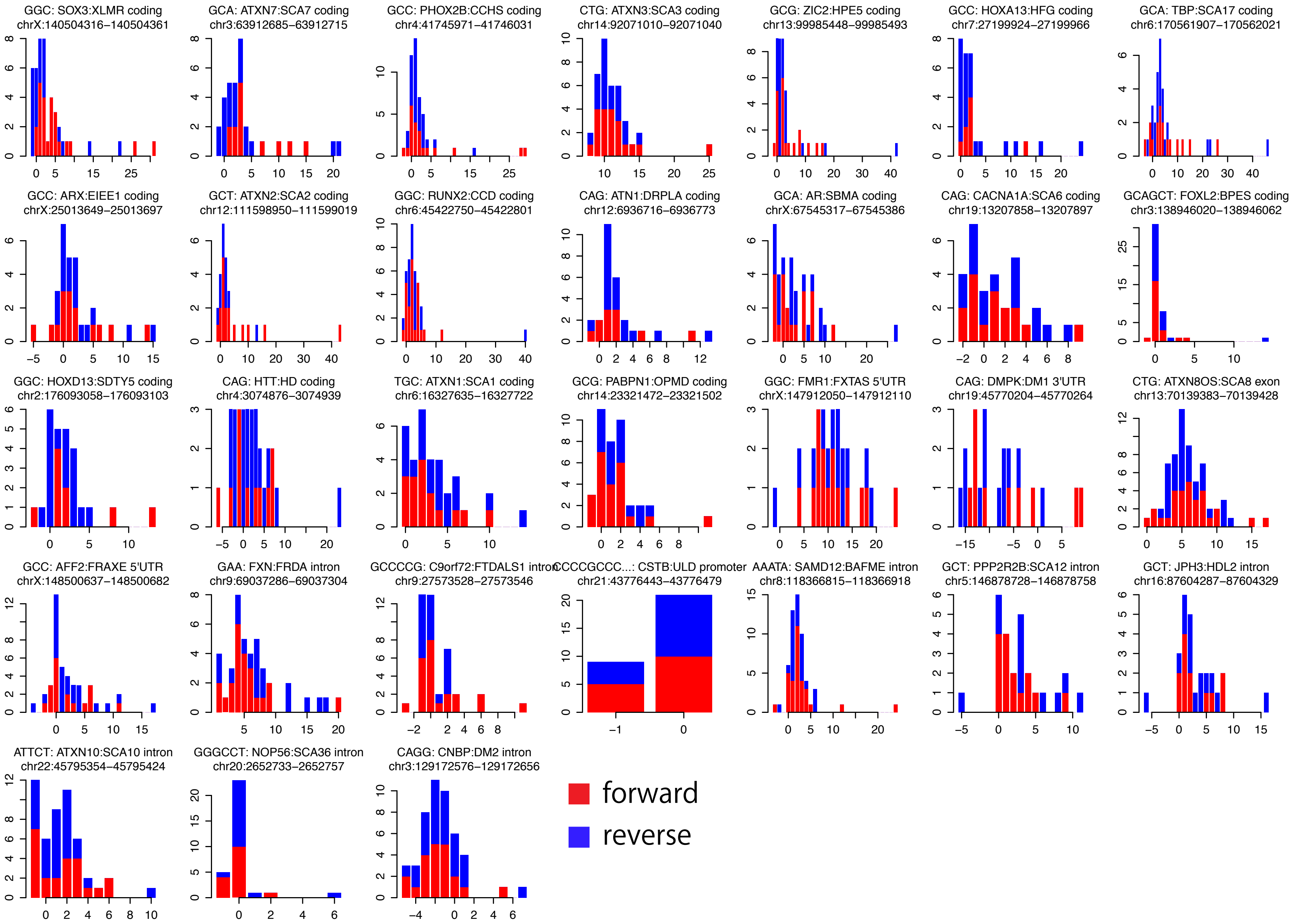

Figure S9

Disease-associated repeats in PacBio sequences for NA12878 (SRR3197748). No obvious pathological expansions were predicted and peaks are around zero except *ATXN3*(~10), *FMR1*(~10), *DMPK*(~-15), and *CNBP*(~-2). There are some outliers in almost all loci, which may be attributed to the PacBio sequencer's insertion prone errors because they were not detected in the nanopore dataset (Figure S8). Forward (red) and reverse strand reads (blue) are shown separately. Histograms are raw output of tandem-genotypes-plot.

Figure S10

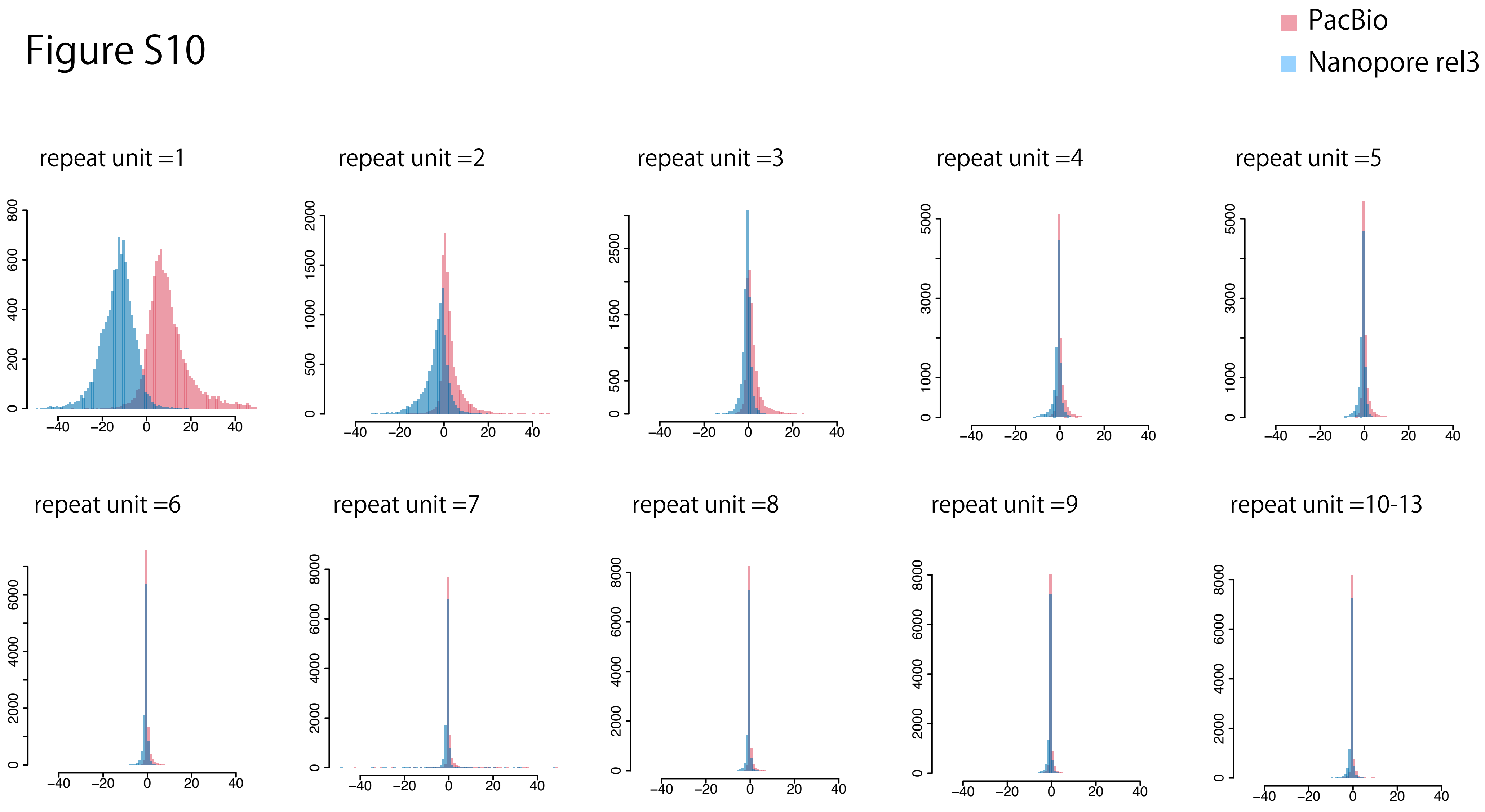

Figure S10

Genome-wide distribution of predicted change in repeat copy number, for PacBio and nanopore (rel3) reads from the same human (NA12878). The distributions are shown separately for different repeat unit lengths. y-axis: read count, x-axis: change in copy number relative to the reference human genome (hg38). Nanopore reads are shown in blue and PacBio in red.

Figure S11

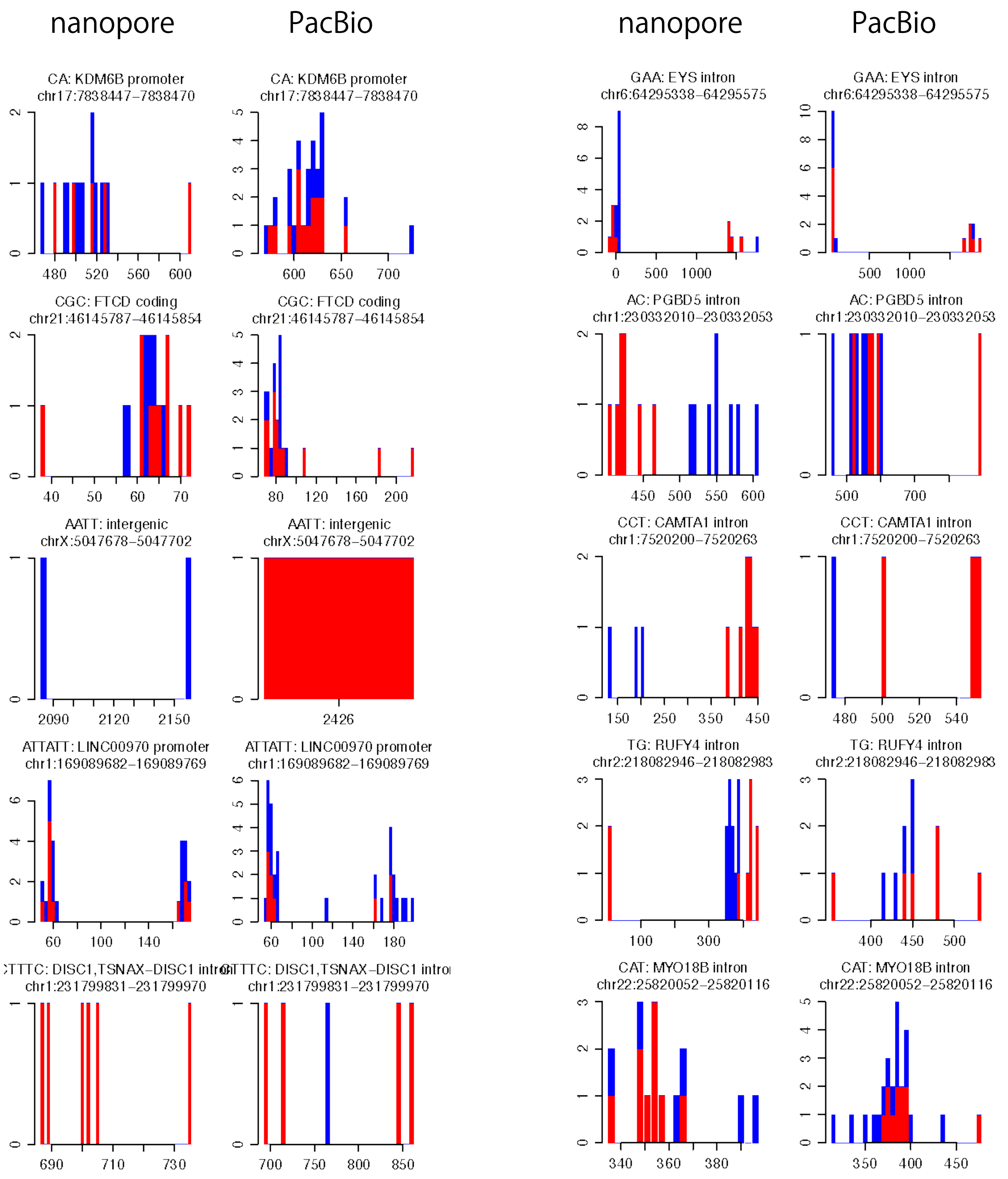

Figure S11

Distribution of predicted change in repeat copy number, in nanopore (rel3, left) and PacBio (SRR3197748, right) reads from NA12878, at ten repeat loci. Forward (red) and reverse strand reads (blue) are shown separately. y-axis: read count, x-axis: change in copy number relative to reference genome hg38. These loci are the 5th, 10th, 15th, 20th, etc. in priority order from the nanopore data.

Figure S12

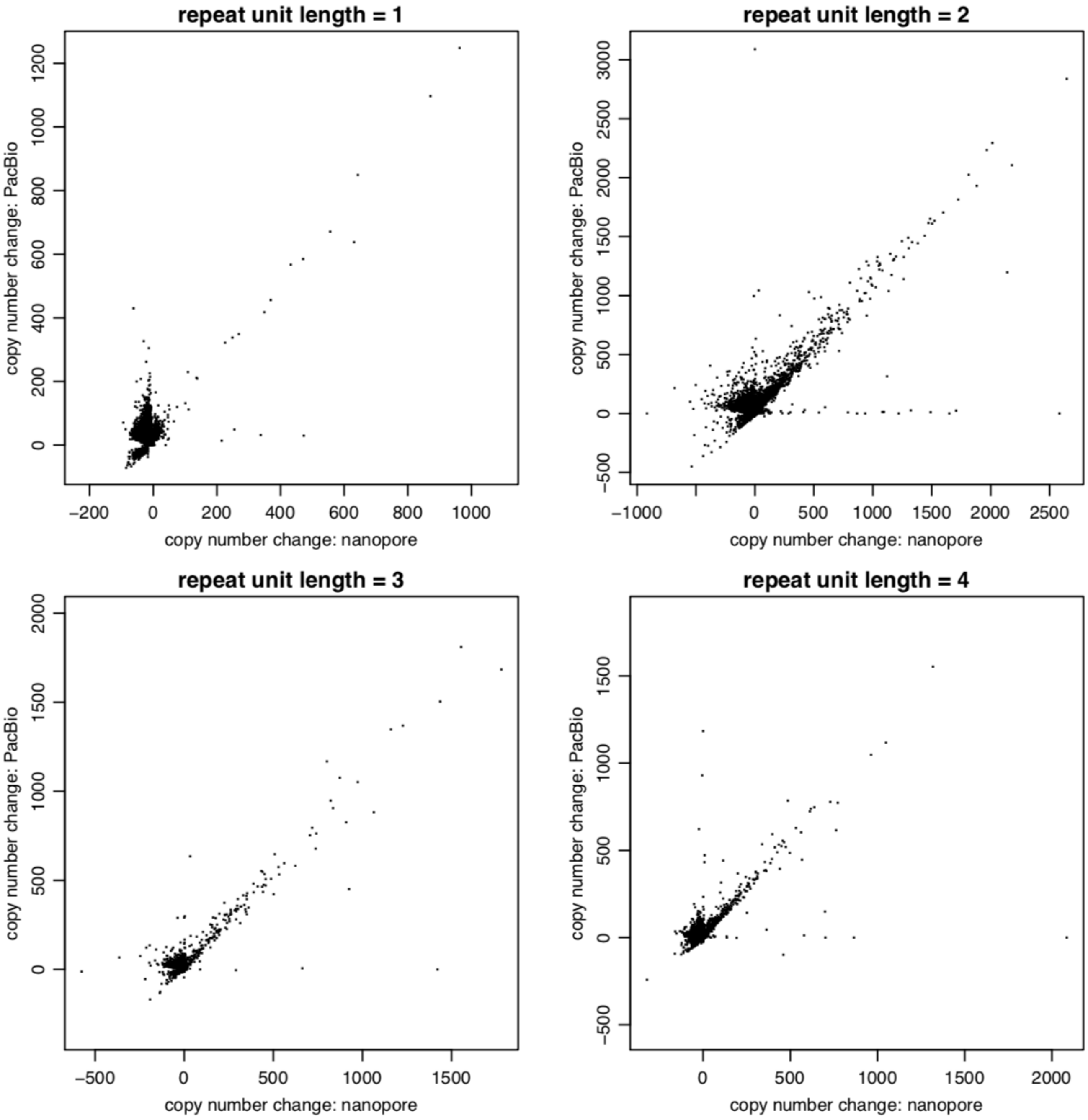

Figure S12

Copy number change (relative to reference genome hg38) of tandem repeats in NA12878, from nanopore (rel3) and PacBio (SRR3197748) reads. Each repeat has multiple predictions of copy-number change, one per DNA read. For each repeat and dataset (nanopore and PacBio), the most extreme expansion and contraction were ignored, and the most extreme remaining change is shown.

Figure S13

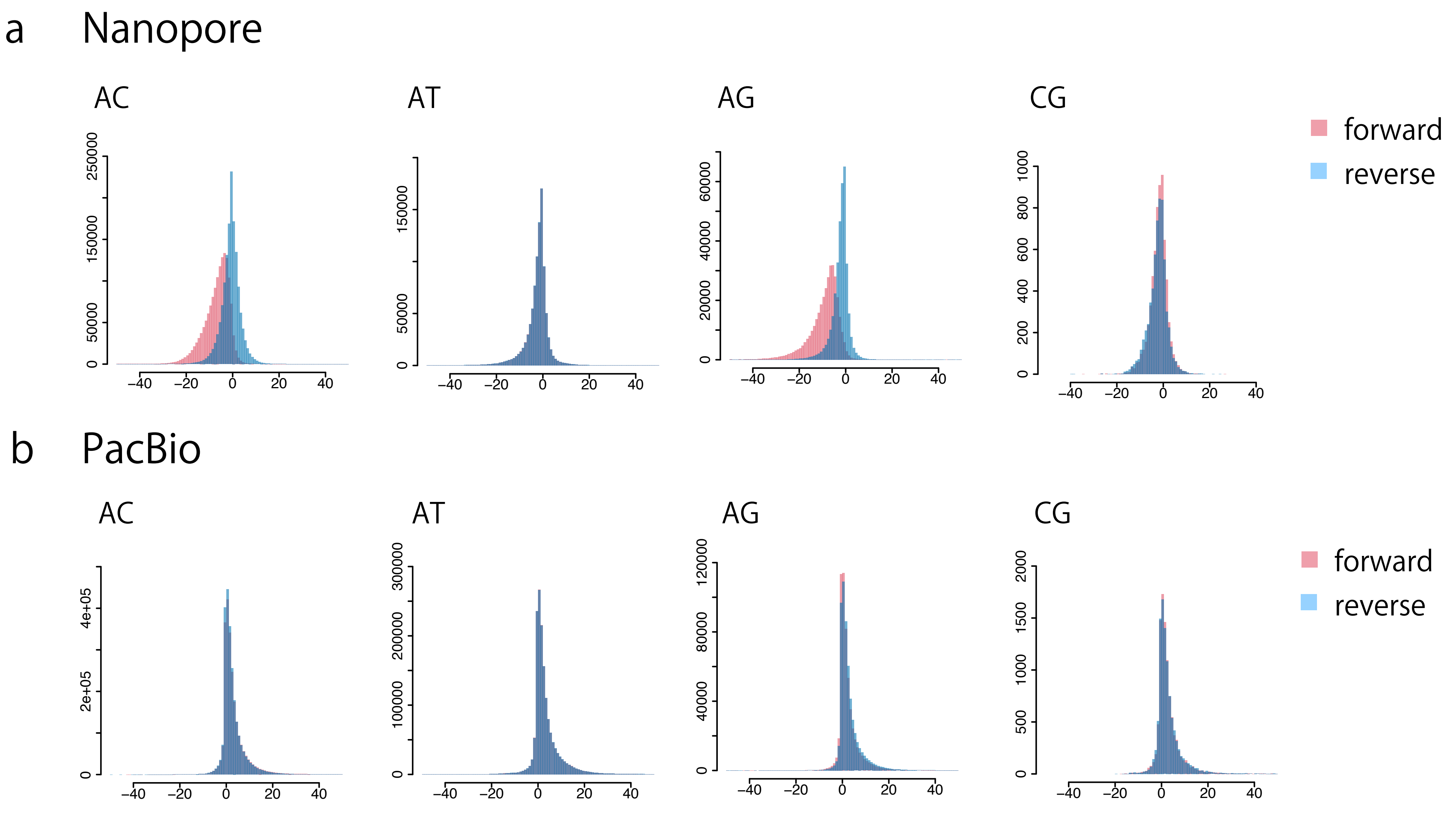

Figure S13

Genome-wide distribution of predicted copy-number change in dinucleotide repeats, for (a) nanopore (rel3) and (b) PacBio reads from the same human (NA12878). The distributions are shown separately for every possible type of dinucleotide repeat. Reads covering the forward strand of each repeat sequence are shown in red, and reads covering the reverse strand in blue. Note that AT and CG are palindromic. y-axis: read count, x-axis: change in copy number relative to the reference human genome (hg38).

Figure S14

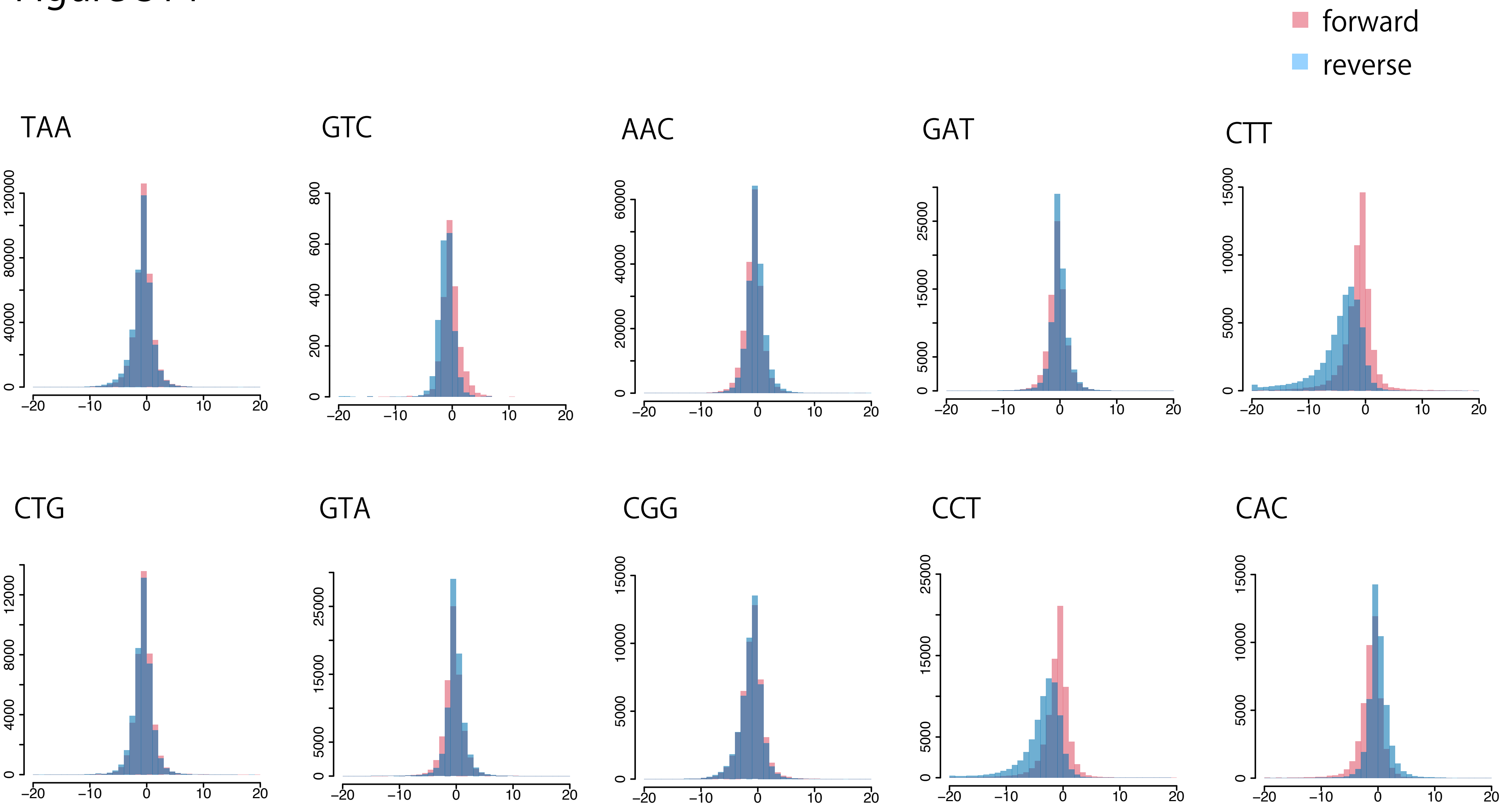

Figure S14
Genome-wide distribution of predicted copy-number change in triplet repeats, for nanopore (rel3) reads from NA12878. The distributions are shown separately for every possible type of triplet repeat. (Note that, for example, TAA includes: TAA, ATA, AAT, TTA, TAT and ATT.) Reads covering the forward strand of each repeat sequence are shown in red, and reads covering the reverse strand in blue. y-axis: read count, x-axis: change in copy number relative to the reference human genome (hg38).

Figure S15

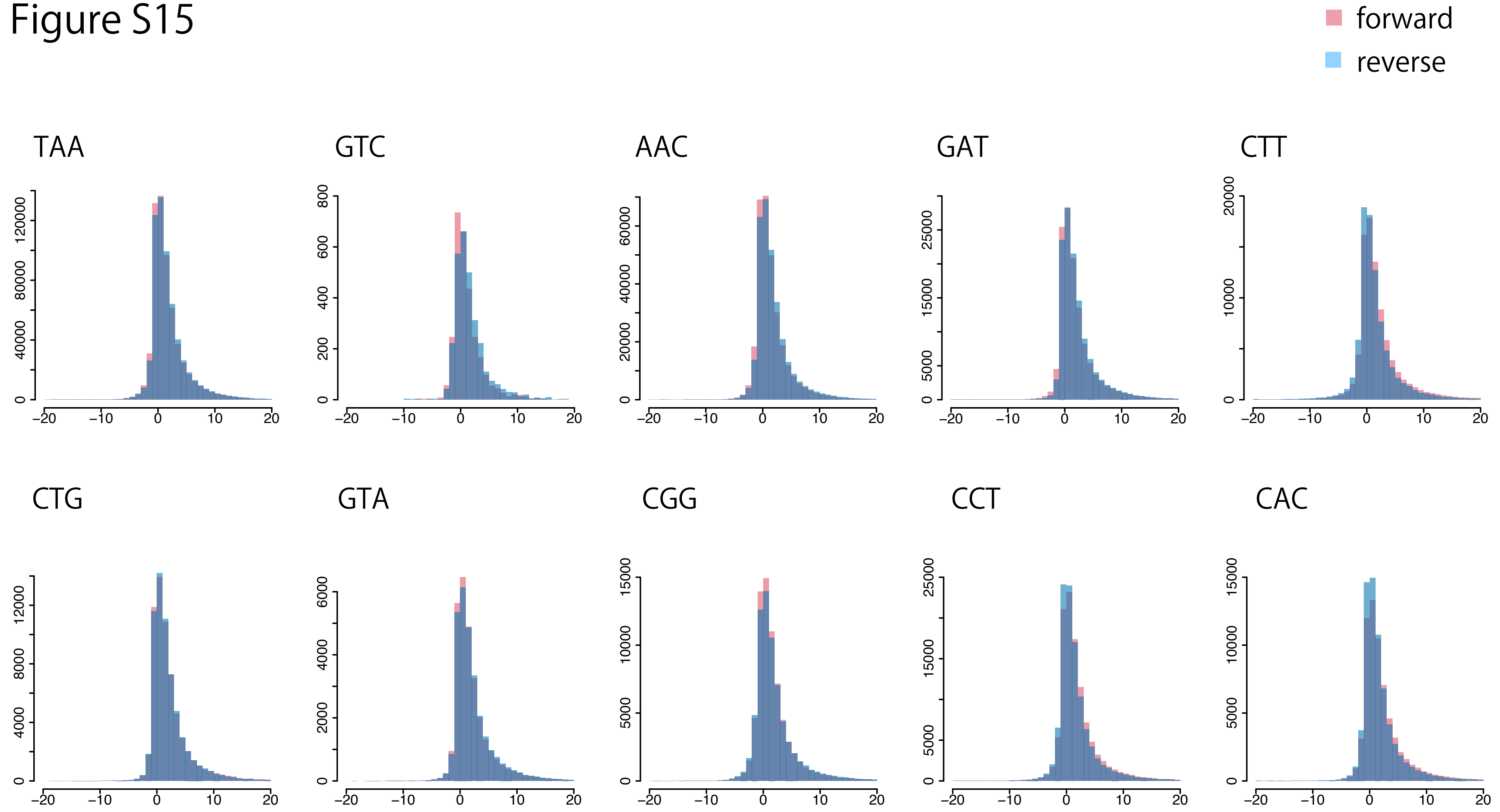

Figure S15
Genome-wide distribution of predicted copy-number change in triplet repeats, for PacBio reads from NA12878. The distributions are shown separately for every possible type of triplet repeat. Reads covering the forward strand of each repeat sequence are shown in red, and reads covering the reverse strand in blue. y-axis: read count, x-axis: change in copy number relative to the reference human genome (hg38).

Figure S16

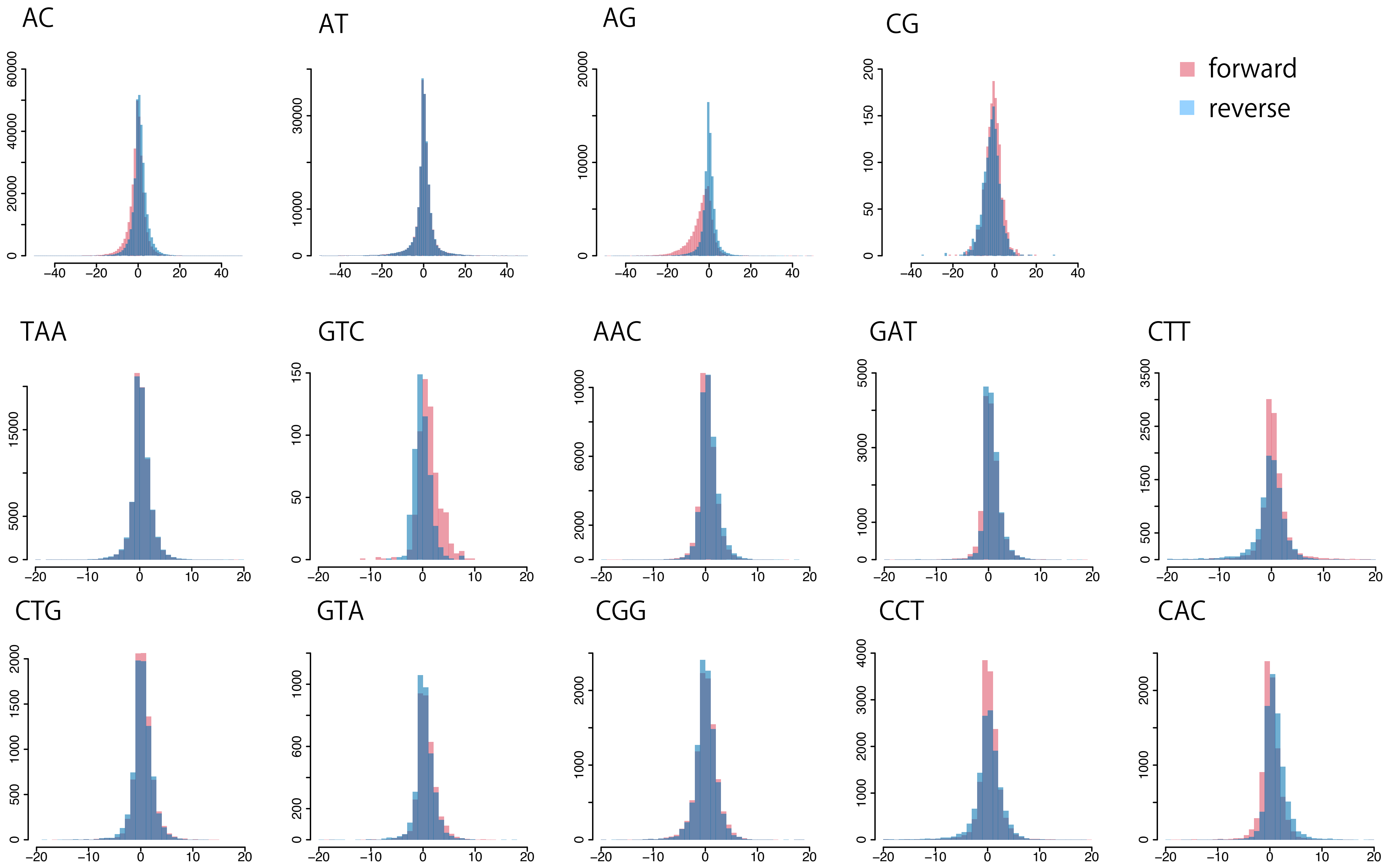

Figure S16

Genome-wide distribution of predicted change in repeat copy number, for nanopore MinION reads basecalled using MinKNOW 1.11.5. The distributions are shown separately for every possible type of di- and tri-nucleotide repeat. Reads covering the forward strand of each repeat sequence are shown in red, and reads covering the reverse strand in blue. y-axis: read count, x-axis: change in copy number relative to the reference human genome (hg38).

Figure S17

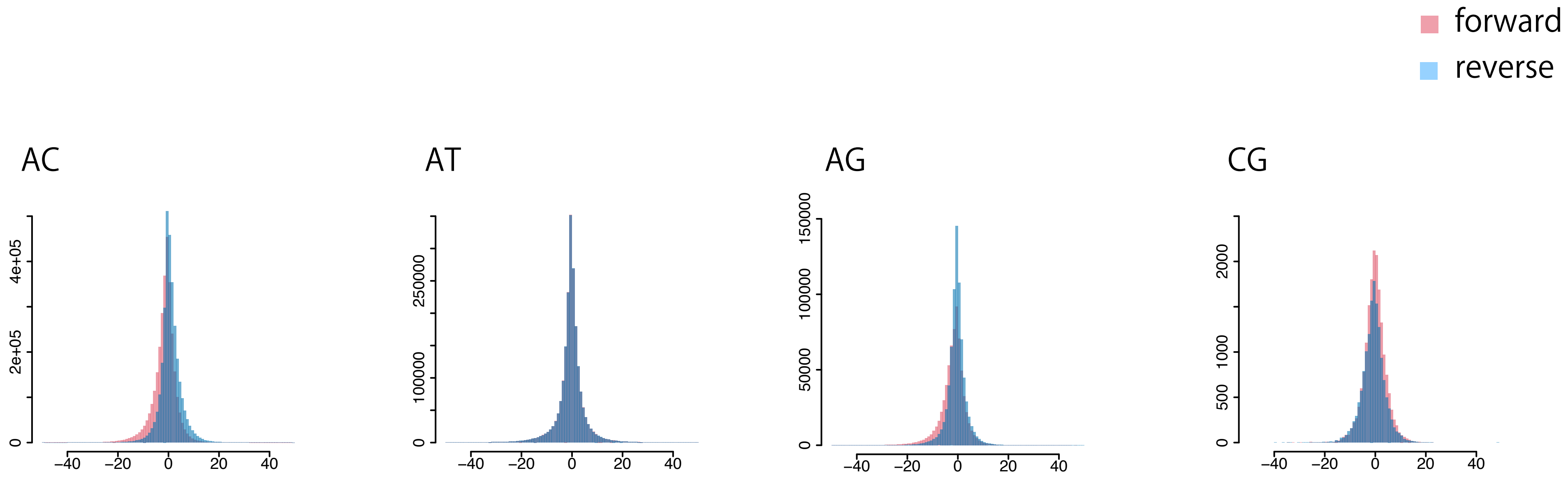

Figure S17

Genome-wide distribution of predicted copy-number change in dinucleotide repeats, for PromethION nanopore reads. The distributions are shown separately for every possible type of dinucleotide repeat. Reads covering the forward strand of each repeat sequence are shown in red, and reads covering the reverse strand in blue. y-axis: read count, x-axis: change in copy number relative to the reference human genome (hg38).

Figure S18

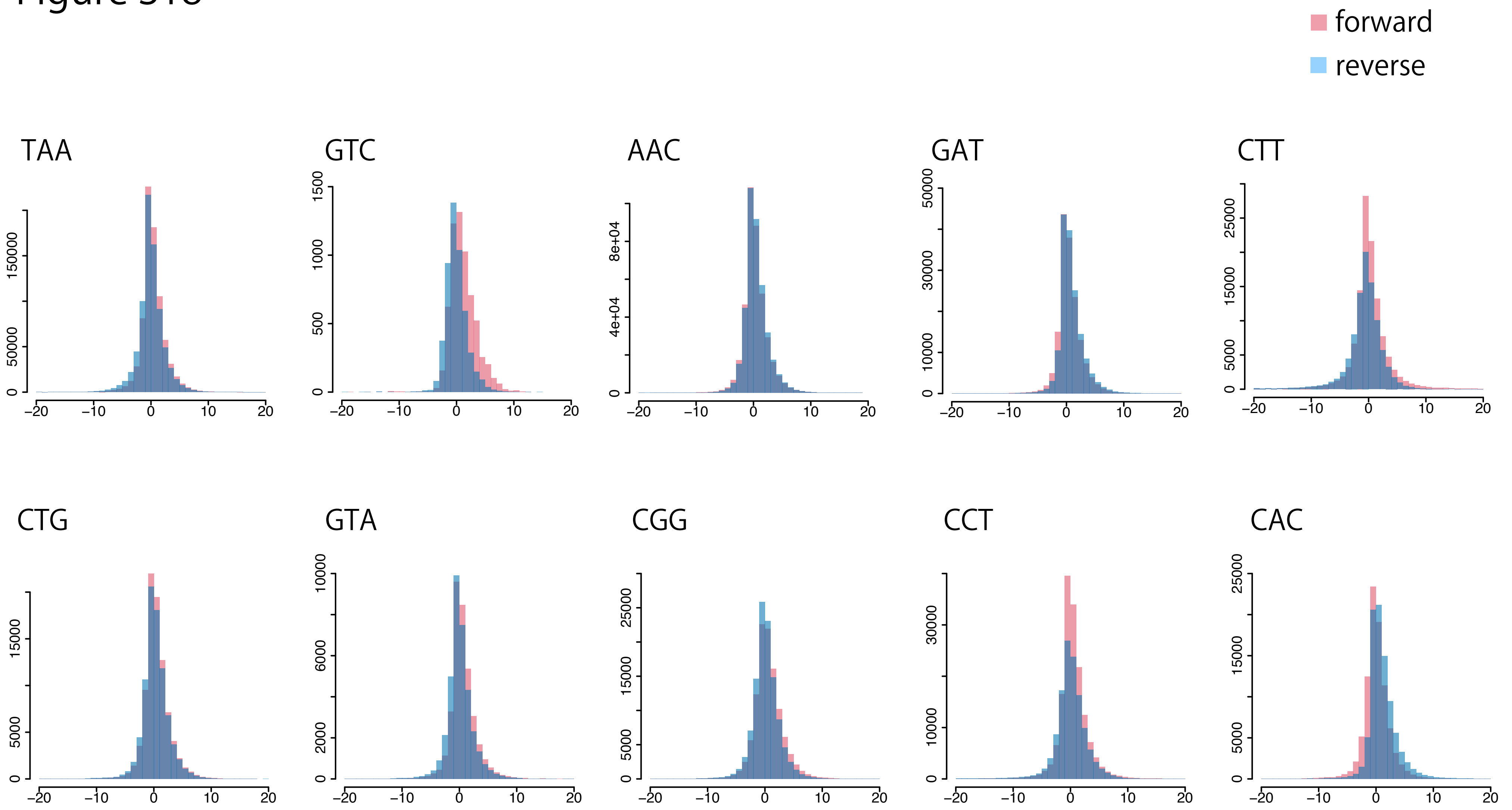

Figure S18

Genome-wide distribution of predicted copy-number change in triplet repeats, for PromethION nanopore reads. The distributions are shown separately for every possible type of triplet repeat. Reads covering the forward strand of each repeat sequence are shown in red, and reads covering the reverse strand in blue. y-axis: read count, x-axis: change in copy number relative to the reference human genome (hg38).

Figure S19

With WindowMasker

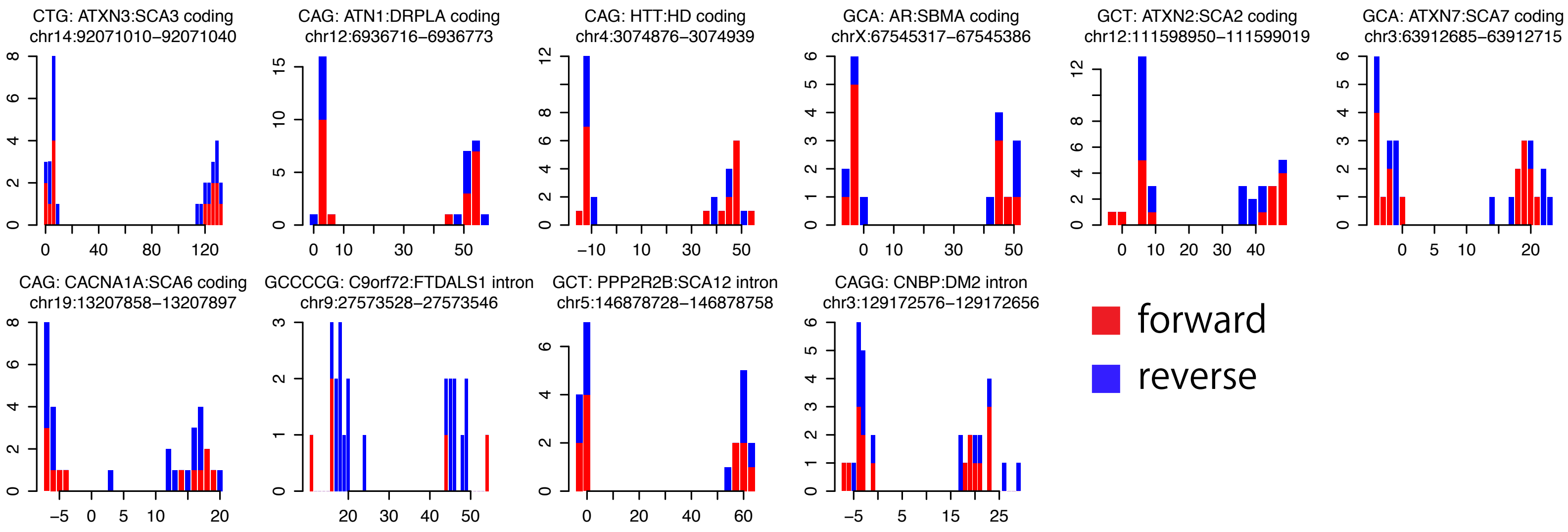

Without WindowMasker

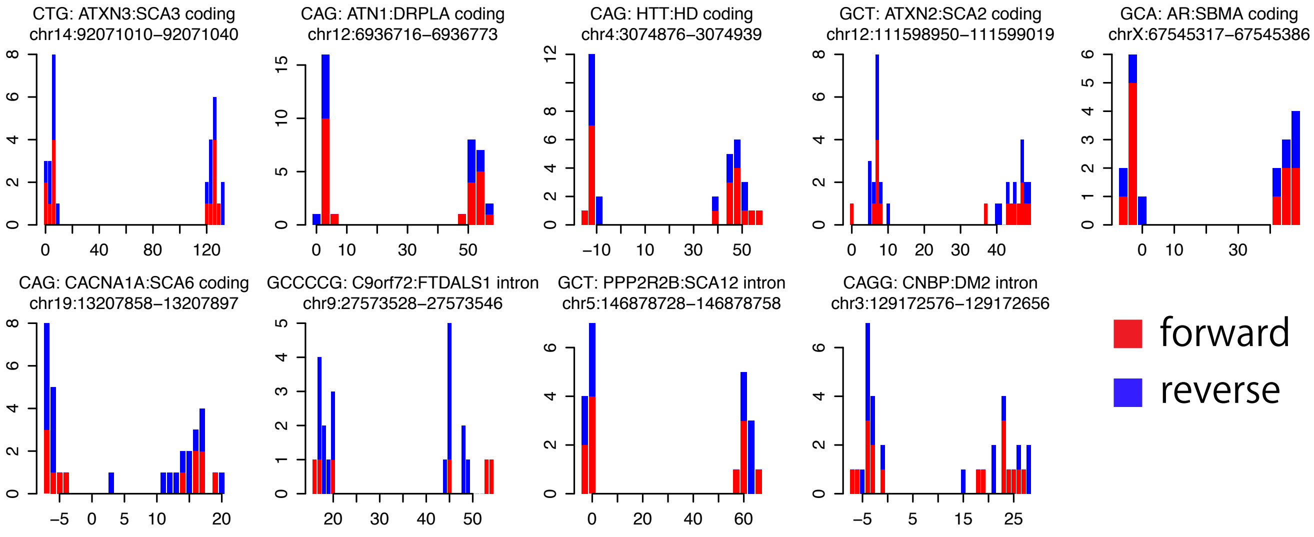

Figure S19

Comparison of tandem-genotypes results for chimeric reads with disease-causing expansions, with or without WindowMasker. Forward (red) and reverse strand reads (blue) are shown separately. Histograms are raw output of tandem-genotypes-plot.

Figure S20

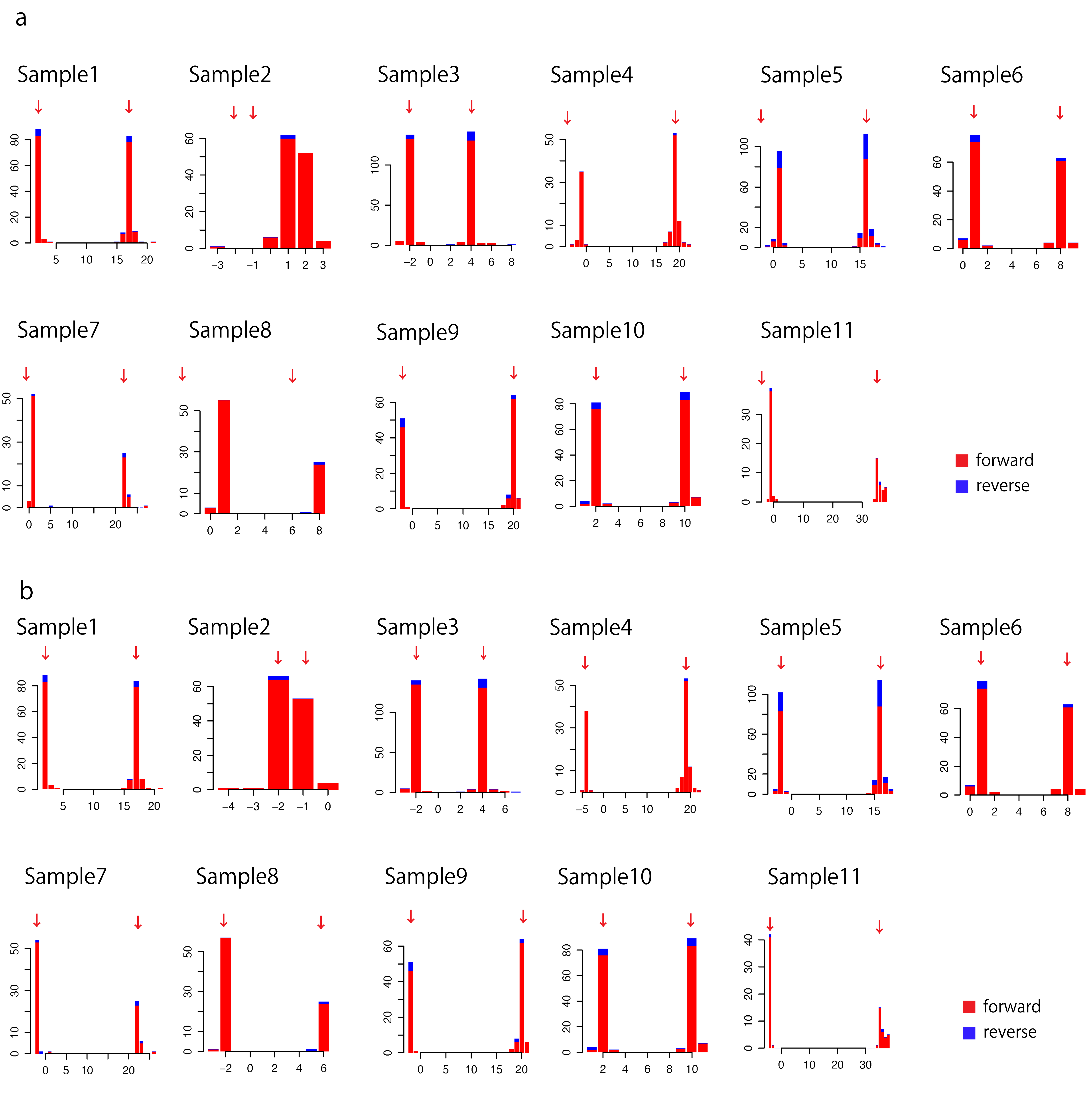

Figure S20
No-Amp datasets of HTT repeat are analyzed by tandem-genotypes without (a) and with (b) - -near=0 option.
y-axis: read count, x-axis: change in copy number relative to the reference human genome.
Red arrows: projected copy number change by Fragment analysis.Forward (red) and reverse strand reads (blue) are shown separately.
These histograms were made by tandem-genotypes-plot.

data	chr	start	end	repeat	gene	tandem-genotypes	NGMLR-sniffles	NanoSV	NGMLR-PBSV
BAFME	chr8	118,366,815	118,366,918	AAATA	*SAMD12*	**detected**	not detected	not detected	not detected
rel3	chr10	54,421,448	54,421,530	TATAT	*PCDH15*	**detected**	not detected	not detected	**detected**
rel3	chr8	48,173,947	48,174,212	GT	intergenic	**detected**	**detected**	not detected	**detected**

Table S1
Lines of output for BAFME: 73,986 (NGMLR–sniffles), 35,485 (PBSV), 33,260 (NanoSV)
Lines of output for rel3: 69,320 (NGMLR–sniffles), 284,870 (PBSV), 54,804 (NanoSV)

		tandem-genotypes			RepeatHMM	PacmonSTR		
		last-train	lastal	tandem-genotypes		blasr	makeBinnedAnchors	PacmonSTR
subject A	real	33m14.835s	15m12.080s	0m3.201	79m19.096s	149m51.665s	0m11.380s	23m2.279s
	user	115m15.589s	80m20.140s	0m2,663	78m41.695s	115m30.293s	0m9.332s	35m51.914s
	sys	9m19.388s	2m9.400s	0m0.051	1m13.406s	27m4.352s	0m0.628s	1m55.797s
subject B	real	40m17.749s	106m23.823s	0m14.713	357m32.226s	838m14.793s	0m51.217s	547m0.712s
	user	138m47.820s	420m49.632s	0m10.504	355m53.665s	781m33.127s	0m49.306s	796m12.366s
	sys	7m33.356s	2m26.925s	0m0.051	3m1.018s	42m55.671s	0m1.220s	81m17.654s
subject C-1	real	17m13.241s	18m26.777s	0m3.396	118m45.216s	201m16.102s	0m21.357s	172m15.222s
	user	55m45.861s	91m30.515s	0m3.173	114m39.965s	167m41.300s	0m19.315s	204m36.091s
	sys	7m20.692s	1m50.115s	0m0.162	4m57.707s	26m26.460s	0m0.994s	50m28.461s
subject C-2	real	19m18.086s	97m5.913s	0m19.980	478m54.832s	549m30.811s	1m23.667s	1285m34.057s
	user	55m15.294s	534m46.885s	0m14.756	478m41.078s	509m55.824s	0m52.368s	1702m1.804s
	sys	8m16.952s	2m40.927s	0m0.289	3m14.759s	30m31.731s	0m1.377s	300m28.184s

Table S2. Computation time for SCA10 datasets aligned to GRCh38. real: wall clock time. user: CPU time. sys: CPU time within the system.

	With WindowMasker			Without WindowMasker		
	last-train	lastal	tandem-genotypes	last-train	lastal	tandem-genotypes
chimeric-reads	real 1m32.584s<br>user 2m34.296s<br>sys 0m47.601s	real 0m37.398s<br>user 0m35.969s<br>sys 0m40.720s	real 0m0.374s<br>user 0m0.331s<br>sys 0m0.019s	real 12m28.813s<br>user 48m20.117s<br>sys 1m15.414s	real 2m12.376s<br>user 6m22.801s<br>sys 0m33.055s	real 0m0.433s<br>user 0m0.415s<br>sys 0m0.015s

Table S3. Computation time for chimeric reads with disease-causing expansion aligned to GRCh38 are compared with or without Windowmasker. real: wall clock time. user: CPU time. sys: CPU time system.

	last-train	lastal	NGMLR
BAFME	real 7m35.726s<br>user 8m25.278s<br>sys 1m9.650s	real 792m14.366s<br>user 5675m34.799s<br>sys 5m43.371s	real 2228m54.286s<br>user 17707m27.325s<br>sys 34m28.691s
rel3	real 15m20.654s<br>user 15m7.098s<br>sys 1m59.262s	real 2460m2.969s<br>user 15292m28.427s<br>sys 15m0.834s	real 4486m17.778s<br>user 35068m16.084s<br>sys 519m18.076s

Table S4. Computation time of alignment for PacBio (BAFME, 15X) and nanopore (rel3, 30X) to whole human genome reference. Reads were aligned to GRCh38 using LAST and NGMLR. real: wall clock time. user: CPU time. sys: CPU time within the system.

	default parameter	random1	random2	random3	random4	random5	random6	random7	random8	random9	random10
coding	50	57	72	35	62	41	26	38	42	61	35
5'UTR	20	21	24	22	16	23	20	15	24	28	23
3'UTR	20	30	18	10	24	11	19	24	25	21	13
exon	15	20	13	17	16	8	13	11	10	20	16
promoter	15	20	13	8	17	17	19	12	15	14	22
intron	5	4	5	6	3	5	7	6	3	5	7

Table S5. Randomly chosen parameters for prioritization.

	BAFME	ATXN3:SCA3	ATN1:DRPLA	HTT:HD	AR:SBMA	ATXN2:SCA2	ATXN7:SCA7	CACNA1A:SCA6
default parameter	4	1	3	4	5	6	7	10
random1	7	1	2	4	5	6	9	10
random2	7	1	2	3	5	6	7	8
random3	3	1	3	5	6	7	9	11
random4	20	1	2	3	4	5	7	10
random5	3	1	3	4	5	6	7	10
random6	2	1	5	7	8	10	12	15
random7	3	1	3	5	6	7	9	12
random8	10	1	2	4	6	7	8	11
random9	6	1	2	4	5	6	8	10
random10	3	1	3	4	6	7	9	13

Table S6. Multi-dataset prioritization of repeat expansions in BAFME patient and protein-coding regions using different random parameters. Numbers represent rank out of 0.7 million [illegible] regions.

name of plasmid	backbone	repeat	restriction enzyme	copy number	reference copy number	Sanger-seq confirmation
EGFP-(CAA)15	pEGFP-C1	CAA	BamHI-HF	15	15	yes
EGFP- (CAA)109	pEGFP-C1	CAA	BamHI-HF	109	15	yes
EGFP-(GGGGCC)52	pEGFP-C1	GGGGCC	NheI	52	3	yes
EGFP-(GGGGCC)21	pEGFP-C1	GGGGCC	NheI	21	3	yes
pBS-(CAG)6	pBS	CAG	DraIII	6	6	yes
RFP-(CTG)18	pEGFP-C1	CAG	EcoRI-HF	18	6	yes
pBS-(CAG)30	pBS	CAG	EcoRI-HF or DraIII	30	6	yes
YFP-(GCT)70	pEYFP-C1	CAG	EcoRI-HF	70	6	yes
YFP-(CAG)130	pEYFP-C1	CAG	BamHI-HF	130	6	yes
pBS-i(CCTG)45	pBS	iCCTG	BamHI-HF	45	15	yes

Table S7

Plasmids used in this study. Enzyme digestion was done using the indicated restriction enzymes. Note that pEGFP-C1 vec
was modified from the original vector and may contain additional sequence.

		PCR product	annotation	gene	region	ref length	primer forward	primer reverse
PCDH15-intron-repeat	chr10:54421448-54421530	588	TATAT	*PCDH15*	intronic	82	GGCCTCTGGAACTATTTTGG	TACGGGACACCTGACACTGA
chr8-intergenic-repeat	chr8:48173947-48174212	359	GT	-	intergenic	265	AGAATTGCCTTGGGCTGAAT	GGTGGAATCCAGGAACTCAA

Table S8. PCR primers.
